# Structural alterations and inflammation in the heart after multiple trauma followed by reamed versus non-reamed femoral nailing

**DOI:** 10.1371/journal.pone.0235220

**Published:** 2020-06-25

**Authors:** Meike Baur, Birte Weber, Ina Lackner, Florian Gebhard, Roman Pfeifer, Paolo Cinelli, Sascha Halvachizadeh, Michel Teuben, Miriam Lipiski, Nikola Cesarovic, Hans-Christoph Pape, Miriam Kalbitz

**Affiliations:** 1 Department of Traumatology, Hand-, Plastic-, and Reconstructive Surgery, Center of Surgery, University of Ulm, Ulm, Germany; 2 Department of Trauma, University Hospital of Zurich, Zurich, Switzerland; 3 Department of Orthopaedic Trauma, RWTH Aachen University, Aachen, Germany; 4 Department of Surgical Research, University Hospital of Zurich, Zurich, Switzerland; 5 Department of Health Sciences, Translational Cardiovascular Technologies, Swiss Federal Institute of Technology, Zurich, Switzerland; John Hunter Hospital and University of Newcastle, AUSTRALIA

## Abstract

**Background:**

Approximately 30,000 patients with blunt cardiac trauma are recorded each year in the United States. Blunt cardiac injuries after trauma are associated with a longer hospital stay and a poor overall outcome. Organ damage after trauma is linked to increased systemic release of pro-inflammatory cytokines and damage-associated molecular patterns. However, the interplay between polytrauma and local cardiac injury is unclear. Additionally, the impact of surgical intervention on this process is currently unknown. This study aimed to determine local cardiac immunological and structural alterations after multiple trauma. Furthermore, the impact of the chosen fracture stabilization strategy (reamed versus non-reamed femoral nailing) on cardiac alterations was studied.

**Experimental approach:**

15 male pigs were either exposed to multiple trauma (blunt chest trauma, laparotomy, liver laceration, femur fracture and haemorrhagic shock) or sham conditions. Blood samples as well as cardiac tissue were analysed 4 h and 6 h after trauma. Additionally, murine HL-1 cells were exposed to a defined polytrauma-cocktail, mimicking the pro-inflammatory conditions after multiple trauma *in vitro*.

**Results:**

After multiple trauma, cardiac structural changes were observed in the left ventricle. More specifically, alterations in the alpha-actinin and desmin protein expression were found. Cardiac structural alterations were accompanied by enhanced local nitrosative stress, increased local inflammation and elevated systemic levels of the high-mobility group box 1 protein. Furthermore, cardiac alterations were observed predominantly in pigs that were treated by non-reamed intramedullary reaming. The polytrauma-cocktail impaired the viability of HL-1 cells *in vitro*, which was accompanied by a release of troponin I and HFABP.

**Discussion:**

Multiple trauma induced cardiac structural alterations *in vivo*, which might contribute to the development of early myocardial damage (EMD). This study also revealed that reamed femoral nailing (reamed) is associated with more prominent immunological cardiac alterations compared to nailing without reaming (non-reamed). This suggests that the choice of the initial fracture treatment strategy might be crucial for the overall outcome as well as for any post-traumatic cardiac consequences.

## Introduction

In the United States, approximately 30,000 patients with blunt cardiac injuries are recorded each year [1]. Heart injury was identified as an independent predictor for a poor outcome after trauma and is associated with a prolonged ventilation interval as well as longer in-hospital stays [2, 3]. Systemic troponin levels were elevated in 43% of trauma patients at admission and correlated with mortality [4]. Furthermore, 78% of patients that were recorded with impaired left ventricular stroke work index died after blunt chest trauma [5]. Therefore, prompt diagnosis and adequate therapy for cardiac damage in is essential in critically injured patients.

Previous experimental studies demonstrated a significant reduction of the cardiac ejection fraction after experimental polytrauma in pigs, followed by fracture fixation either with an external fixator or by intramedullary nailing [6]. The functional impairment was accompanied by increased cardiac damage markers as troponin and HFABP, as well as changes in structural proteins of the heart (alpha-actinin, desmin and connexin 43). Although an inflammatory component of the traumatic cardiac damage was expected, there were no differences between the treatment groups detectable. Currently, the impact of early fracture fixation strategies in polytrauma (namely intramedullary reaming enhanced nailing) on cardiac homeostasis is unclear.

Intramedullary nailing with reaming might reduce the occurrence of fracture non-union [7, 8]. However, reaming has been associated with an increased blood loss and a longer duration of operative procedures compared to non-reamed intramedullary nailing [7, 8]. Furthermore, a process of intravasation of bone marrow and fat into the venous blood system [9], followed by the development of an acute respiratory distress syndrome (ARDS), was described after reaming [10]. During reaming a higher volume of embolization has been detected through echocardiographic analysis, compared to intramedullary nailing without reaming [11, 12]. Besides causing possible distress to the lungs, embolization was also associated with multiple organ failure [13]. Therefore, the choice of the applied therapeutic approach might be highly relevant with regard to cardiovascular consequences after long bone fractures especially in the context of multiple trauma [13].

Because of their cardio-depressive effects, pro-inflammatory cytokines and damage-associated molecular patterns (DAMPs) such as the high-mobility group box 1 (HMGB-1) protein and extracellular histones have been linked to post-traumatic cardiac dysfunction [6, 14–16]. A trend towards higher systemic concentrations of IL-6, plasma elastase, CD11b expression and soluble intercellular adhesion molecule 1 (s-ICAM-1) was found after femoral reaming, compared to non-reamed fracture stabilization [17]. Additionally, the stimulatory capacity of polymorphonuclear leukocytes rose in patients with reamed fractures [18]. Activated neutrophils were also detected in the heart after blunt chest trauma and might therefore play a role in post-traumatic cardiac damage [16].

Since complex myocardial impairment after multiple trauma in pigs has been described previously, [6] the aim of the current study was to compare the effects of reaming versus unreamed intramedullary nailing for unilateral femur fractures in the context of multiple injury with regard to the development of early myocardial damage (EMD). The EMD is defined as the damage of cardiomyocytes in the early phase after trauma and is associated with a systemic increase of cardiac damage markers, as well as with an impairment in diastolic and systolic function of the heart. In this study, we focused on the hearts’ local inflammatory reaction and structural changes.

## Materials and methods

### Animals

This study presents partial results obtained from a large animal porcine multiple trauma model, conducted by the TREAT research group.

All procedures conformed to the Society of Laboratory Animal Science (GV-SOLAS) as well as the National Animal Welfare Law and after approval by the responsible government authority (Kanton Zürich, Gesundheitsdirektion Veterinäramt) and performed according to the guidelines of the Federation of European Laboratory Animal Science Association (FELASA). 15 male pigs weighing 40–60 ± 5 kg (*Sus scrofa domestica*) were included in the study (mean height: 123,6 cm). General instrumentation, anaesthesia and trauma induction were described previously by Horst *et al*. [19]. Animals were held in controlled environment with 21 ± 3°C room temperature (50% humidity), with a light/dark cycle of 12 h. Water was available for animals *ad libitum*.

### Multiple trauma in pigs

Pigs were randomized into a multiple trauma (n = 10) and the sham (n = 5) group. Multiple trauma consisted of a chest trauma, laparotomy, liver laceration, femur fracture as well as a hemorrhagic shock (ISS ≥ 27). A bolt gun (Blitz-Kemen, turbocut JOBB GmbH, Germany) with cattle-killing cartridges (9x17; DynamitNobel AG; Troisdorf, Germany) was used to apply the femur fracture by positioning it on the mid third of the right femur. On the right dorsal lower chest, a pair of panels (Steel 0.8 cm, lead 1.0 cm thickness) was placed to introduce chest trauma. The shock wave was also generated by a bolt gun as described before [20, 21]. The right upper liver lobe was explored during midline-laparotomy. For the penetrating hepatic injury, a cross-like incision through half of the liver tissue was performed. Liver package followed 30 sec of uncontrolled bleeding. Afterwards the animals underwent pressure-controlled and volume-limited hemorrhagic shock for 60 min due to the withdrawal of blood until they reached a mean arterial pressure (MAP) of 25 ± 5 mmHg with a maximum withdrawal amount of 45% of the total blood volume. With the help of established trauma guidelines (ATLS®, AWMF-S3 guideline on Treatment of Patients with Severe and Multiple Injuries®) the animals were resuscitated after the shock period. Their FiO_2_ was adjusted and their withdrawn blood as well as fluids were given (Sterofundin ISO^®^, 2 ml/kg body weight/h). Normothermia (38.7–39.8°C) was tried to be reached. Sham animals (n = 5, sham) received instrumentation and anesthesia but neither trauma nor hemorrhage. The treatment also included the stabilization of the femur fracture. This was done either through femoral nailing without reaming (n = 5, non-reamed) or reaming (n = 5, reamed). If the femoral nailing group is mentioned, it will be referring to the animals that received femoral nailing without reaming.

### Follow-up, euthanasia and sample collection

After an observation period of 6 h, pigs were euthanized with Na-Pentobarbital. Tissue of the heart’s left ventricle was obtained 6 h after trauma. To differentiate between different layers of the myocardium, we fixed superficial and luminal layers of the ventricle separately with 4% formalin. Additionally, tissue was quick frozen in liquid nitrogen for analysis of mRNA and protein expression. At baseline, 4 h and 6 h after multiple trauma blood samples were taken.

### Connexin 43 ELISA

To quantify systemic levels of connexin 43 (Cx43) the Pig GJA1 / CX43 / Connexin 43 ELISA Kit (LifeSpan BioSciences Inc., Seattle, WA, USA) was utilized. The assay was performed according to manufacturer’s instructions.

### HFABP ELISA

The mouse cardiac FABP ELISA (Life Diagnostics, West Chester, PA, USA) was used for measurement of heart fatty acid binding protein (HFABP) concentration in the supernatant of murine HL-1 cells in presence of a defined polytrauma-cocktail (PTC). The assay was performed to manufacturer’s instructions.

### HMGB-1 ELISA

Using the HMGB-1 ELISA (IBL International GmbH, Hamburg, Deutschland) systemic amounts of HMGB-1 were measured. The assay was performed according to manufacturer’s instructions.

### Histone ELISA

The Cell Death Detection ELISA^PLUS^ (Roche Diagnostics, Indianapolis, IN, USA) was used to quantify systemic levels of extracellular histones. The assay was performed according to manufacturer’s instructions.

### Troponin I ELISA

To measure the amount of troponin I in the supernatant of HL-1 cells treated with a polytraumacocktail, the Ultra-Sensitive Mouse Cardiac Troponin-I ELISA Kit (Life Diagnostics, West Chester, PA, USA) was used. The assay was performed according to manufacturer’s instructions.

### General procedure for staining paraffin-embedded left ventricle

The tissue was deparaffinised and rehydrated in ethanol. To unmask potential binding sites the sections were given into citrate buffer (pH = 6) at 100°C. Afterwards unspecific binding sites were blocked through incubation with 10% goat serum.

### Immunofluorescence (IF) staining of the left ventricle

For specific antigen binding the tissue was incubated with the respective antibody for alpha-Actinin (GeneTex, Irvine, CA, USA) and desmin (GeneTex, Irvine, CA, USA) for 1 h at room temperature. By using either AlexaFluor488-labelled- (Jackson Immuno Research Laboratory, West Grove, PA, USA) or AlexaFluor647-labelled- (Jackson Immuno Research Laboratory, West Grove, PA, USA) secondary antibodies the specific antigen antibody complex was made visible. The cell nuclei were stained with Hoechst 33342 (Sigma-Aldrich, St. Louis, MO, USA). Sections were analysed by using an Axio Imager M.1 microscope and the Zeiss AxioVision software 4.9 (Zeiss, Jena, Germany). Results are presented as mean fluorescence intensity.

### Immunohistochemistry (IHC) staining of the left ventricle

By incubating the sections for 1 h at room temperature with the respective primary antibodies for connexin 43 (Cx43) (CellSignaling Technology, Danvers, MA, USA) and nitrotyrosine (Merckmillipore, Darmstadt, Germany) specific antigen binding was performed. For detecting the specific antigen antibody complex Dako REAL™ Detection System (Dako, Glostrup, Denmark) was used. The cell nuclei were counterstained with Hematoxylin. Results are presented as pixel density.

### Western blot

First tissue of the left ventricle was homogenized by using RIPA Lysis and Extraction Buffer (ThermoFisher Scientific, Waltham, MA, USA) as well as Phenylmethanesulfonyl fluoride, Protease Inhibitor Cocktail and Sodium orthovanadate (all three from Sigma-Aldrich, St. Louis, MO, USA). With Pierce™ BCA Protein Assay Kit (ThermoFisher Scientific, Waltham, MA, USA) the protein concentration in homogenates was measured. For electrophoresis the homogenates were loaded onto a 4–20% Mini-PROTEAN^®^ TGX Stain-Free™ Protein Gel (BioRad, Hercules, CA, USA) and afterwards transferred with a Trans-Blot^®^ Turbo™ Transfer System using Mini PVDF Transfer Packs (both from Bio-Rad, Hercules, CA, USA). Membranes were blocked with 5% milk for 1,5 h at room temperature and afterwards incubated with the respective primary antibody for connexin 43 (Cx43) (CellSignaling Technology, Danvers, MA, USA) and IL-6 (abcam, Cambridge, UK) overnight at 4°C. After washing, an anti-rabbit IgG HRP-linked antibody (CellSignaling Technology, Danvers, MA, USA) was used as secondary antibody for 1 h at room temperature. The blots were analyzed with ChemiDoc (Bio-Rad, Hercules, CA, USA) and the ImageLab software (version 5.2, Bio-Rad). Results are presented as intensity.

### Cell viability assay

Cell viability was analysed using Cell Titer-Glo® Luminescent Cell Viability Assay (Promega, Madison, WI, USA). Cells were treated with a defined polytrauma-cocktail (PTC), comprising 10 ng/ml activated complement factor 5 (C5a), 500 ng/ml activated complement factor 3 (C3a), 250 pg/ml Interleukin 1 beta (IL-1β), 500 pg/ml Interleukin 6 (IL-6), 150 pg/ml Interleukin 8 (IL-8), 10 ng/ml tumour necrose factor (TNF), 100 ng/ml High Mobility Group Box Protein-1 (HMGB-1) and 20 μg/ml extracellular histones for 6 h at 37°C and 5% CO_2_ [22]. All procedures were performed according to manufactures instructions. For all experiments n = 6. Results are presented as counts per second.

### RNA isolation

For qPCR experiments, murine HL-1 cells (Merck, Darmstadt, Germany) were treated with PTC for 6 h at 37°C and 5% CO_2_. RNA isolation from cell lysates was performed by using ISOLATE II RNA Mini Kit (Meridian Bioscience, Cincinnati, OH). Remaining DNA was digested by DNase I (Meridian Bioscience, Cincinnati, OH). Both procedures were performed according to manufacturer’s instructions.

RNA was extracted from quick frozen tissue of the pigs’ left ventricle by using Invitrogen TRIzol Reagent (Sigma-Aldrich, St. Louis, MO, USA). Afterwards Invitrogen DNAse I Amplification Grade (ThermoFisher Scientific, Waltham, MA, USA) was used to digest the remaining DNA. Both procedures were performed according to manufacturer’s instructions.

### Reverse transcribed quantitative polymerase chain reaction (RT-qPCR)

The respective mRNA samples were reverse transcribed in cDNA using SuperScript™ IV VILO™ MasterMix (Invitrogen, Carlsbad, CA). The PowerUp™ SYBR™ Green Master Mix (Applied Biosystems, Waltham, MA) was used for quantitative PCR. All procedures were performed according to the manufacturer’s instructions. For qPCR itself the QuantStudio3 system (Applied Biosystems, Waltham, MA) was utilized.

The used primers are listed in the attachment. Result are presented as fold change.

### Statistical analysis

All values were expressed as means ± SEM. The animal data were analysed by one-way ANOVA followed by Dunnett’s or Tukey’s multiple comparison test. The *in vitro* data were analysed by unpaired student t-test. p≤0.05 was considered as statistically significant. For statistical analysis the GraphPad Prism 7.0 software was used (GraphPad Software, Incorporated, San Diego, CA, USA).

## Results

### Alterations of connexin 43 distribution

**Fig 1:**
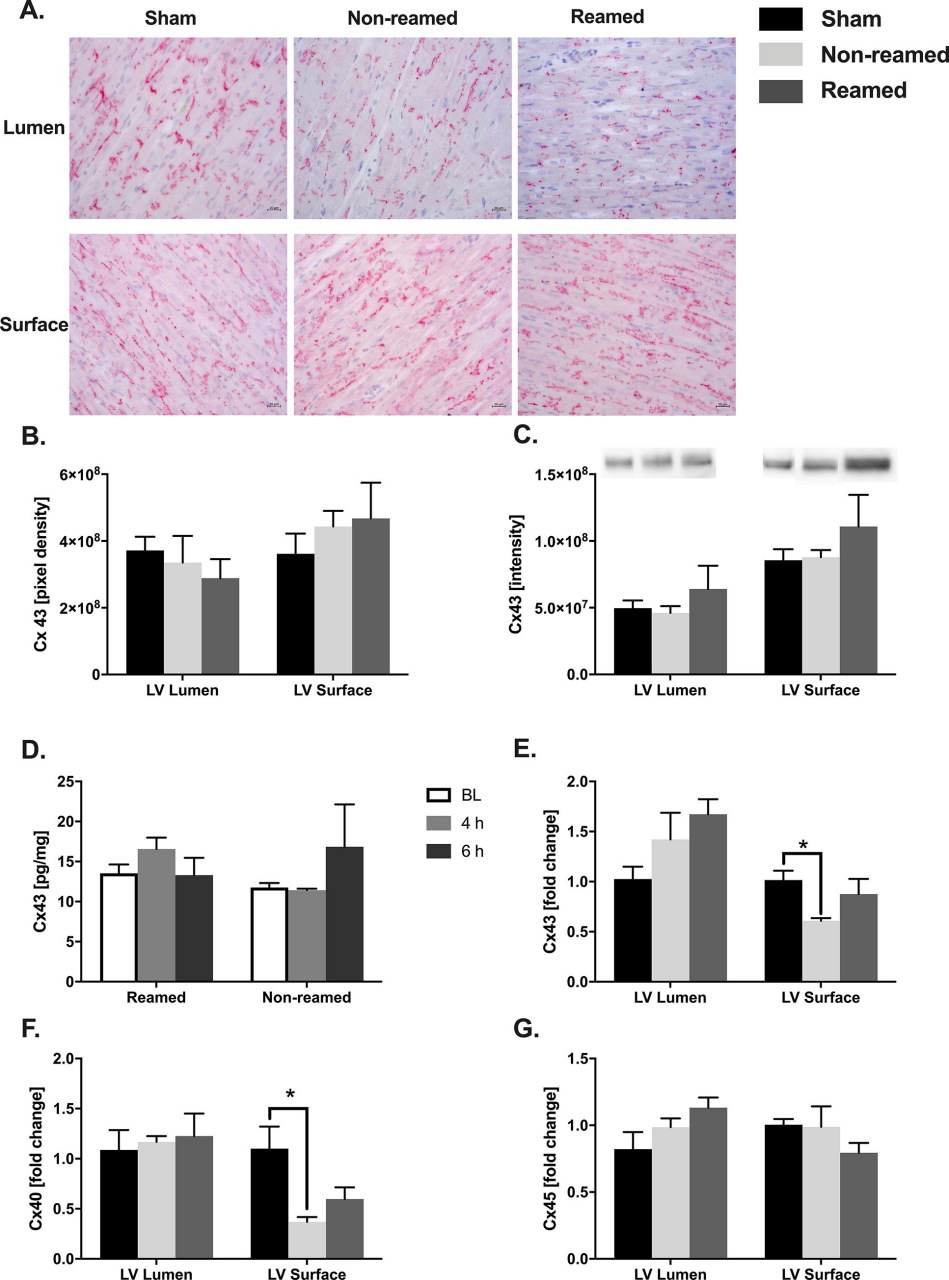
Alteration of connexin 43 distribution after multiple trauma in pigs. A) Representative images of connexin 43 (Cx43) in the superficial and luminal layer of the left ventricle (LV) after multiple trauma and either nailing (non-reamed) or reaming. B) Amount of Connexin 43 measured in stained sections of the cardiac tissue after multiple trauma in pigs. Control/sham animals were presented as black bars, pigs after multiple trauma and nailing (non-reamed) were presented as light grey and pigs after multiple trauma and reaming (reamed) were presented as dark grey bars. C) Western blot analysis of Cx43 protein expression, as well as representative western blot bands. D) Systemic levels of connexin 43 in pg/mg measured in serum taken at baseline (BL), 4h and 6h after trauma. Baseline amounts are represented as white bars, 4 h after trauma as medium grey bars and 6 h after trauma as dark grey bars. E) Cx43 mRNA-expression in the LV presented as fold change. F) Connexin 40 (Cx40) mRNA expression detected in tissue homogenates of the left ventricle 6h after multiple trauma. G) Connexin 45 (Cx45) mRNA expression presented as fold change compared to sham. Results are significant (*) p<0.05. For statistical analysis one-way ANOVA was used. Graphical presentation as mean ± SEM. For each experiment n = 5.

Connexin 43 (Cx43) is a cardiac gap junction protein, which plays an important role in the electromechanical communication of cardiomyocytes. After multiple trauma, Cx43 was no longer strictly expressed at the Z-discs but was translocated from the membrane of the cardiomyocytes into the cytoplasm of the cells (representative images Fig 1A). We did not observe any changes in the protein amount of Cx43 (Fig 1B and 1C). To exclude Cx43 distribution into the circulation, the systemic concentrations of Cx43 in serum were measured at baseline, 4 h as well as 6 h after trauma. No significant differences were found (Fig 1D). A slight increase of Cx43 mRNA expression in the luminal layer of the left ventricle after trauma was recorded. Furthermore, the superficial Cx43 mRNA expression was significantly reduced in pigs after femoral nailing (non-reamed) compared to sham treated animals (Fig 1E).

We further investigated local cardiac alterations in the mRNA expression of Cx40 and Cx45. Although there was no alteration in the Cx40 mRNA expression after multiple trauma in the luminal part of the left ventricle, a significant decrease was shown superficially after femoral nailing without reaming (non-reamed) compared to the sham-treated animals (Fig 1F). No significant differences were found in Cx45 mRNA expression after multiple trauma (Fig 1G).

### Structural changes

**Fig 2:**
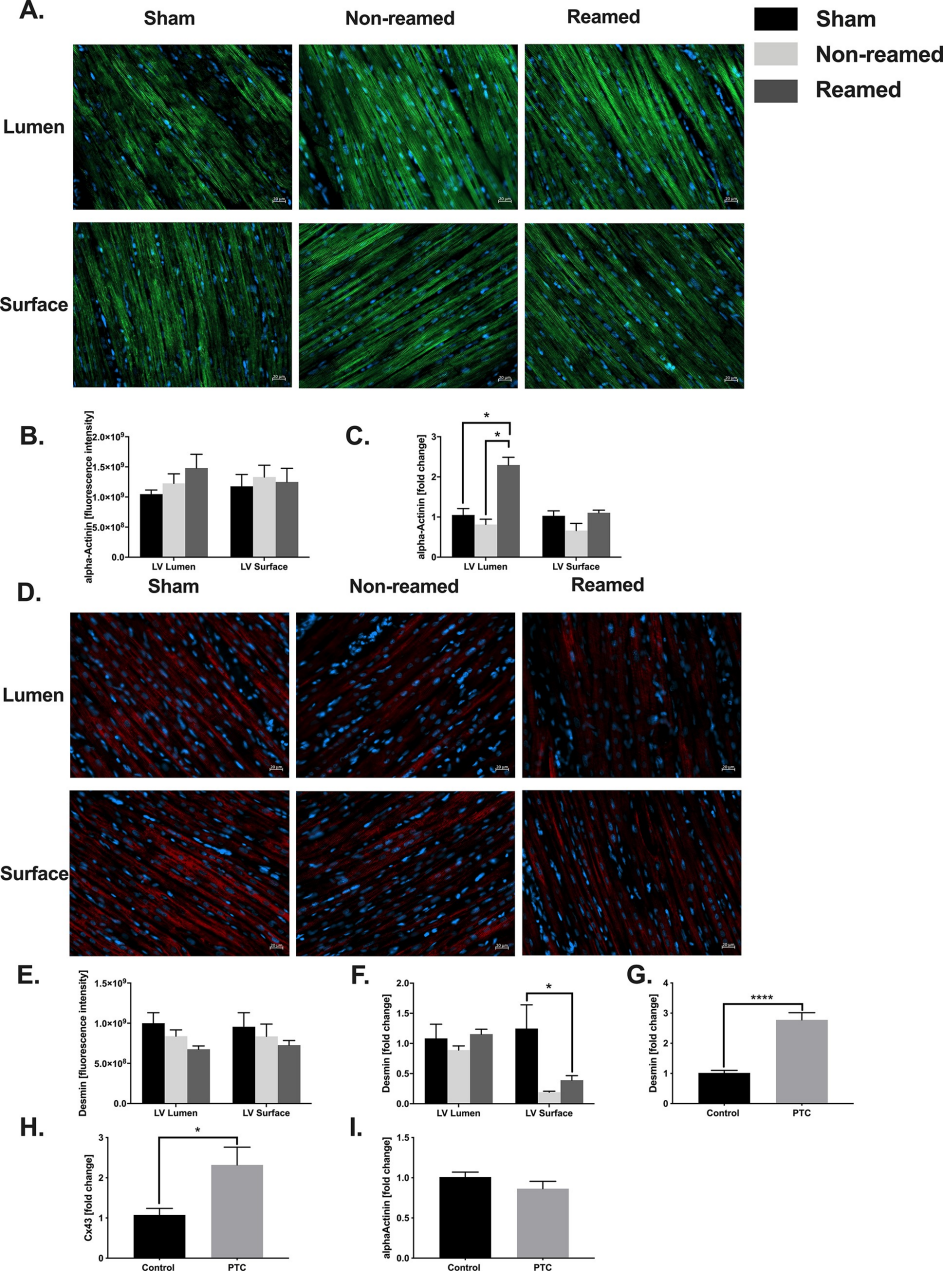
Structural changes of the heart after multiple trauma. A) Representative images of immunofluorescence staining of alpha actinin in the superficial and luminal layer of the left ventricle (LV) after multiple trauma and either nailing (non-reamed) or reaming (reamed) compared to sham. B) Quantitative analysis of alpha-actinin staining. Control/sham animals were presented as black bars, pigs after multiple trauma and nailing (Poly Nail, non-reamed) were presented as light grey and pigs after multiple trauma and reaming (reamed) were presented as dark grey bars. C) mRNA expression of alpha actinin presented as fold change compared to sham. D) Representative images of desmin immunofluorescence staining. E) Results of quantitative analysis of desmin staining in the superficial and luminal layer of left ventricles after multiple trauma. F) Desmin mRNA expression presented as fold change. G) Desmin mRNA expression in HL-1 cells in presence of PTC. H) Cx43 mRNA expression of HL-1 cells after incubation with PTC. I) mRNA expression of alpha-actinin in HL-1 cells after incubation with a defined polytrauma cocktail. Results are significant (*) p<0.05. For statistical analysis one-way ANOVA was used. Graphical presentation as mean ± SEM. For each experiment n = 5.

In order to determine the effects of multiple trauma on cardiac structure and on the cytoskeleton of cardiomyocytes, the expression of alpha-actinin (representative images Fig 2A) and desmin (representative images Fig 2D) were analysed. Luminal, alpha-actinin protein levels were slightly increased in the reaming group compared to sham procedure (Fig 2B). The mRNA expression was significantly increased after reaming in the luminal part of the left ventricle compared to both sham and non-reamed conditions (Fig 2C). In contrast, the amount of desmin decreased slightly after multiple trauma (Fig 2E). The desmin mRNA expression significantly decreased in the superficial part of the left ventricle in the group with reaming compared to the sham group (Fig 2F).

To further confirm our results, *in-vitro* testing was performed and murine cardiomyocytes (HL-1 cells) were incubated with a defined polytrauma cocktail. The mRNA expression of the structure protein desmin significantly increased in presence of the PTC as well as the mRNA expression of Cx43 (Fig 2G and 2H). No changes occurred in the α-actinin mRNA expression (Fig 2I).

### Cardiac inflammation

**Fig 3:**
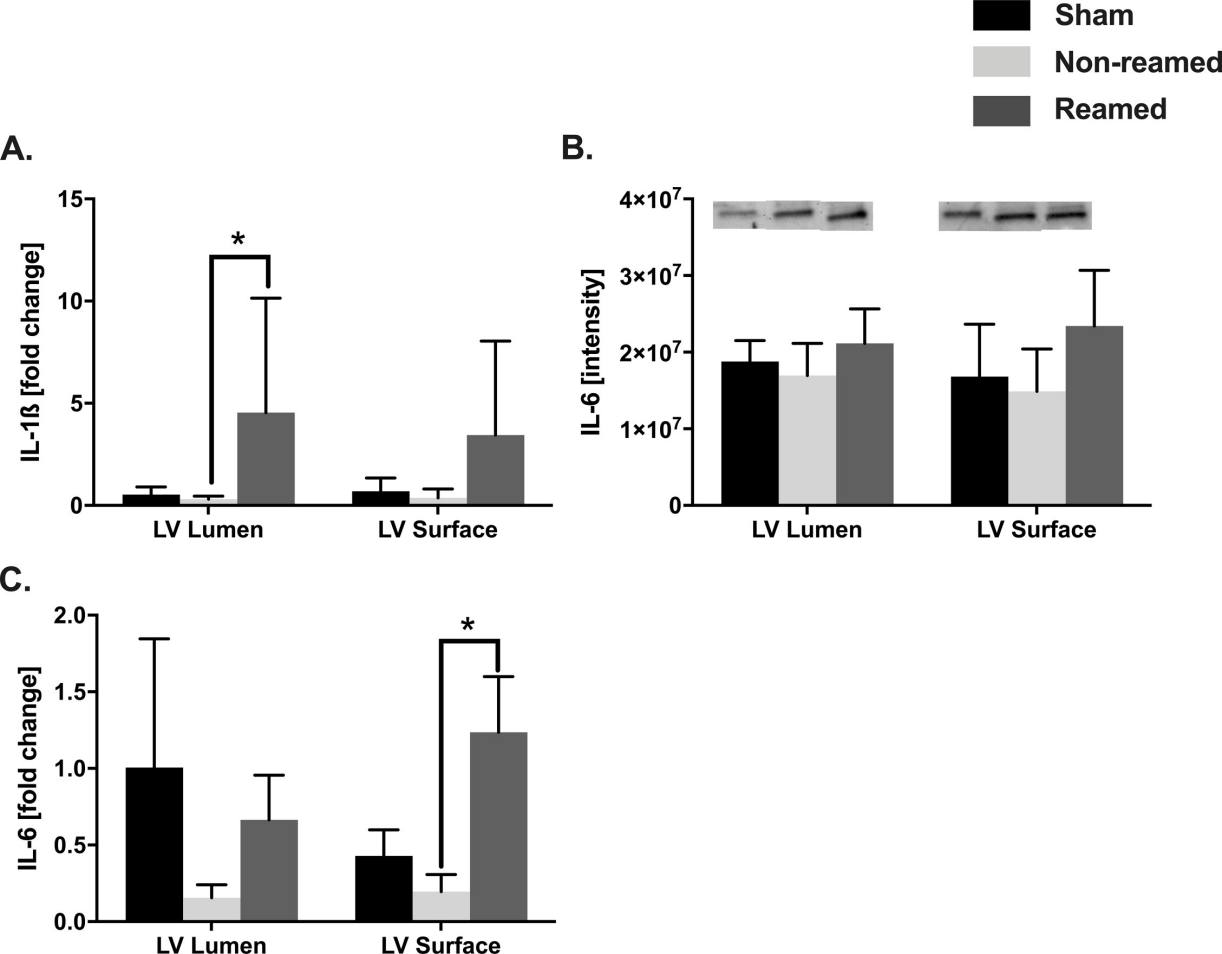
Cardiac inflammation. Control/sham animals were presented in black bars, pigs after multiple trauma and nailing (non-reamed) were presented in light grey and pigs after multiple trauma and reaming (reamed) were presented as dark grey bars. A) mRNA expression of interleukin 1-ß (IL-1ß) of the luminal and superficial layer of the left ventricle, detected by RT-qPCR presented as fold change. B) Interleukin-6 (IL-6) protein expression and representative western blot bands. C) mRNA expression of IL-6 in the left ventricle. n = 5 for each experiment. Results are significant (*) p<0.05. For statistical analysis one-way ANOVA was used. Graphical presentation as mean ± SEM.

To determine cardiac inflammation after multiple trauma, the protein levels as well as the local mRNA expression of pro-inflammatory cytokines were measured in tissue of the left ventricle 6 h after trauma. Increased IL-1β mRNA expression was found in the luminal part of the left ventricle in pigs exposed to reaming, compared to pigs which were treated with unreamed femoral nailing (non-reamed) (Fig 3A). The protein expression of the pro-inflammatory cytokine IL-6 was not increased after trauma (Fig 3B). Compared to the sham animals, pigs with multiple trauma and reaming showed enhanced corresponding mRNA expression of IL-6 in the superficial part of the left ventricle (Fig 3C).

### Nitrosative stress, cardiac tissue damage, apoptosis and DAMPs

**Fig 4:**
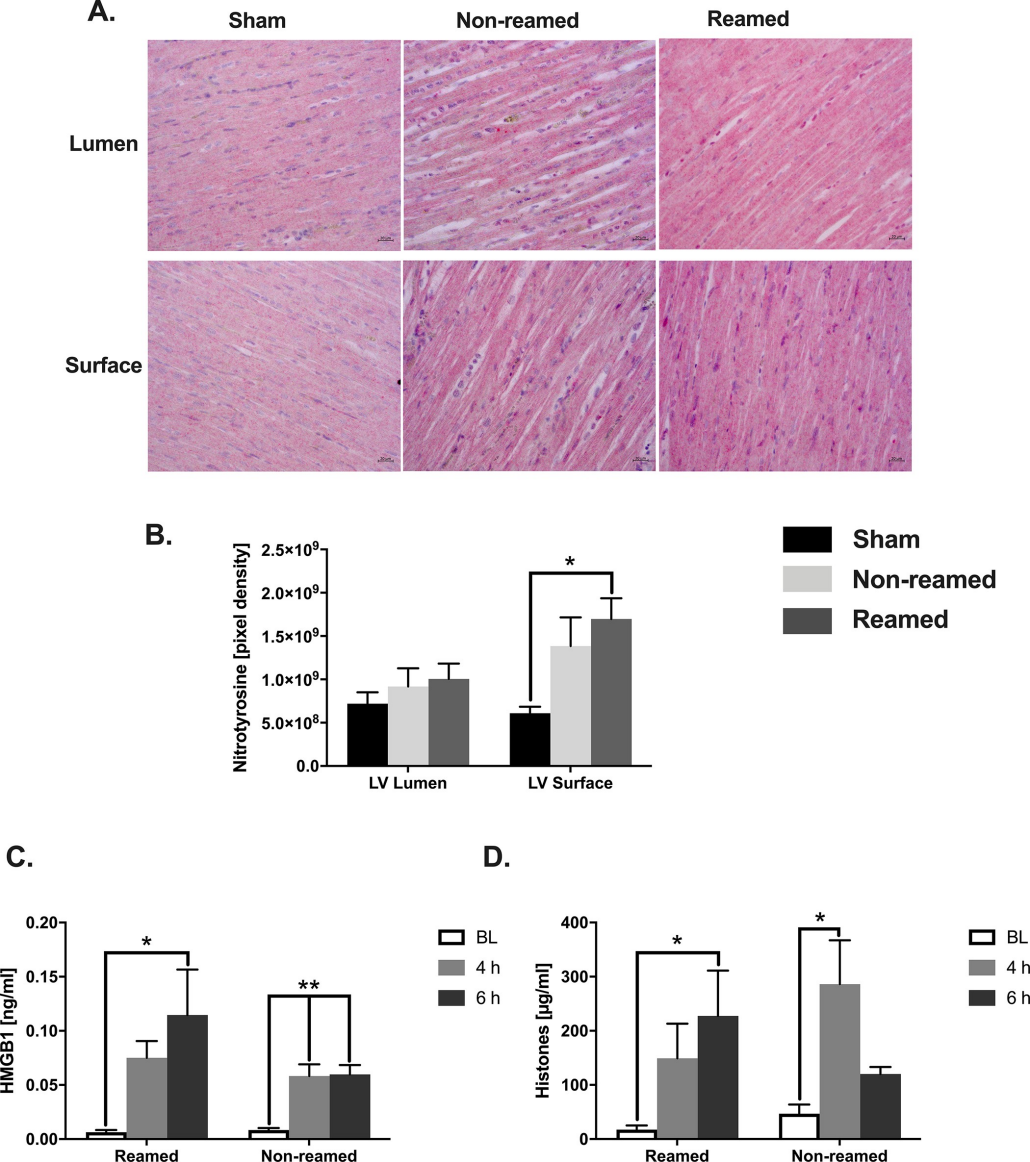
Nitrosative stress, cardiac tissue damage and apoptosis. A) Representative images of the nitrotyrosine staining of the superficial and luminal layer of the left ventricle (LV) after multiple trauma and either nailing (non-reamed) or reaming (Poly Conv, reamed) compared to sham. B) Quantitative analysis of nitrotyrosine staining of the LV. Control/sham animals are presented as black bars, pigs after multiple trauma and nailing (Poly Nail, non-reamed) are presented as light grey and pigs after multiple trauma and reaming (reamed) are presented as dark grey bars. C) Systemic levels of HMGB1 in ng/ml measured by ELISA taken at baseline (BL), 4 h and 6 h after trauma. Baseline amounts are represented as white bars, 4 h after trauma as medium grey bars and 6 h after trauma as dark grey bars. D) Systemic levels of extracellular histones in μg/ml measured by ELISA at baseline (BL), 4 h and 6 h after trauma. Baseline amounts are represented as white bars, 4 h after trauma as medium grey bars and 6 h after trauma as dark grey bars. In each group n = 5. Results are significant (*) p<0.05. For statistical analysis one-way ANOVA was used. Graphical presentation as mean ± SEM.

To analyse local nitrosative stress, the expression of nitrotyrosine was determined in left ventricles after multiple trauma (representative images Fig 4A). As shown in Fig 4B, the amount of nitrosylated tyrosine increased significantly in the superficial part of the left ventricle of pigs, which received reaming compared to sham-treated animals.

The High mobility group box 1 protein (HMGB-1) is considered as a damage-associated molecular pattern (DAMP). While the systemic levels of HMGB-1 increased significantly only after femoral nailing (non-reamed) 4 h after multiple trauma compared to baseline, both trauma groups showed a significant increase of systemic HMGB-1 6 h after trauma (Fig 4C). Systemic extracellular histones increased significantly 6 h after reaming as well as 4 h after femoral nailing (non-reamed) compared to baseline (Fig 4D).

### Calcium balance

**Fig 5:**
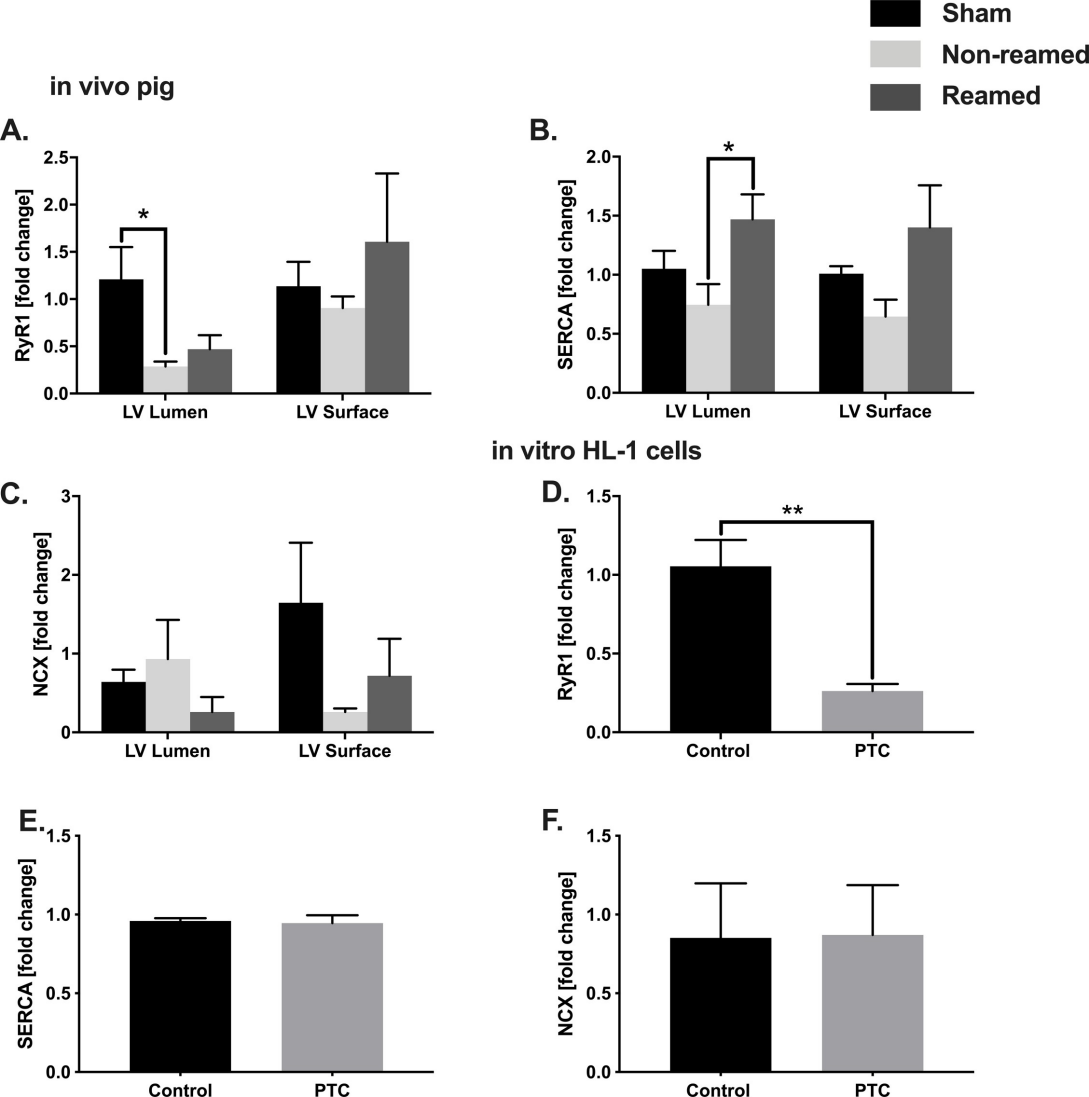
Calcium balance in vivo and in vitro. A) mRNA expression of Ryanodine receptor (RyR1) in tissue homogenates of the superficial and luminal layer of the left ventricle after multiple trauma in pigs. Control/sham animals were presented as black bars, pigs after multiple trauma and nailing (non-reamed), were presented as light grey and pigs after multiple trauma and reaming (reamed) were presented as dark grey bars. B) Sarcoplasmic/ endoplasmic reticulum ATPase (SERCA) mRNA expression after multiple trauma in pigs presented as fold change. C) mRNA expression of natrium calcium exchanger (NCX) presented as fold change. D) RyR1 mRNA expression of HL-1 cells in presence of polytrauma cocktail (PTC). E) SERCA mRNA expression of HL-1 cells incubated with PTC compared to control cells presented as fold change. F) mRNA expression of NCX in HL-1 cells in presence of PTC presented as fold change. Results are significant (*) p<0.05. For statistical analysis one-way ANOVA was used. Graphical presentation as mean ± SEM. For in vitro analysis students t-test was used (in vivo n = 5, in vitro n = 6).

We analysed post-traumatic changes in calcium-handling proteins. The ryanodine receptor 1 (RyR1) mRNA expression significantly decreased in the luminal part of the heart in the group which received femoral nailing (reamed) compared to the sham group (Fig 5A). Furthermore, the sarcoplasmic/endoplasmic reticulum ATPase (SERCA) mRNA expression increased significantly in the reaming group (reamed) in the luminal layer of the left ventricle compared to the nailing group (non-reamed) (Fig 5B). No significant changes in mRNA expression of the sodium-calcium exchanger (NCX) were detectable after multiple trauma in both locations of the left ventricle (Fig 5C).

In an additional set of experiments, we investigated *in vitro* the effects of a defined PTC on the expression of calcium handling proteins in murine HL-1 cells. The mRNA expression of the RyR1 significantly decreased in presence of PTC (Fig 5D), while there was no change in the SERCA mRNA expression (Fig 5E). No changes were observed in the mRNA expression of the NCX in presence of polytrauma cocktail (Fig 5F).

### *In vitro* experiments

**Fig 6:**
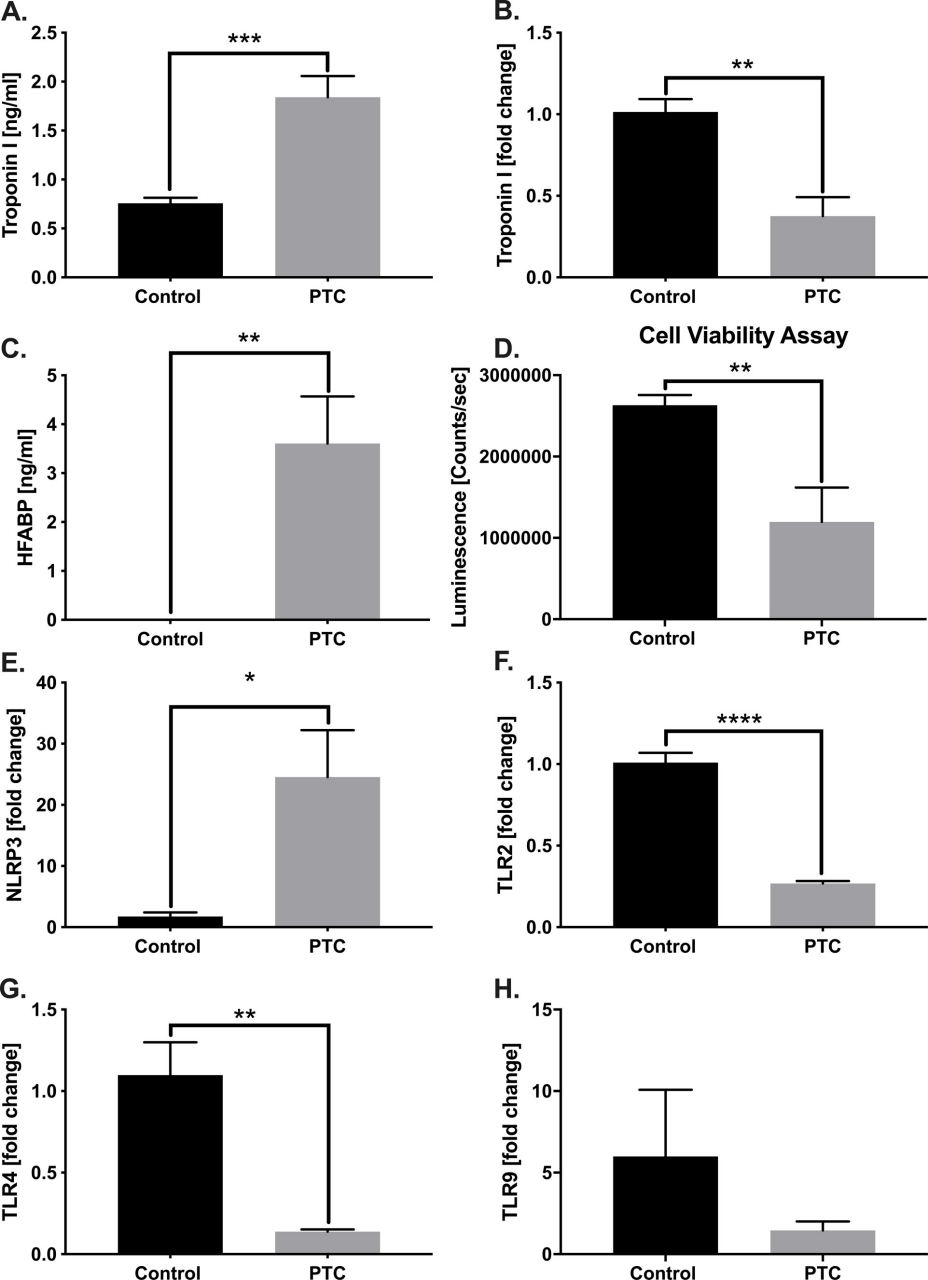
In vitro analysis of HL-1 cells treated with polytrauma cocktail (PTC). A) Troponin I level in supernatant of HL-1 cells in presence of PTC presented as ng/ml. B) mRNA expression of troponin I presented as fold change. C) Amount of heart fatty acid binding protein (HFABP) in supernatant of HL-1 cells after incubation with PTC presented as ng/ml. D) Cell viability in counts/sec in presence of PTC compared to control (PBS). E) mRNA expression of NLRP3 detected by RT-qPCR presented as fold change. mRNA expression of TLR-2 (F), -4 (G) and -9 (H) in presence of PTC presented as fold change. Results are significant (*) p<0.05. Graphical presentation as mean ± SEM. For statistical analysis students t-test was used. For all experiments n = 6.

To further confirm our *in vivo* results, we treated murine HL-1 cells with a defined PTC, mimicking post-traumatic inflammatory conditions. To assess whether the mouse cardiomyocytes release troponin I in the presence of PTC, the protein levels were measured in the supernatant. There was a significant increase of troponin I in the supernatant of HL-1 cells in the presence of PTC (Fig 6A). Moreover, the troponin I mRNA expression significantly decreased after incubation with the PTC (Fig 6B). As another heart specific damage marker, the heart fatty acid binding protein was also tested and significantly increased levels were found in supernatant of HL-1 cells in presence of PTC (Fig 6C). Additionally, cell viability significantly decreased in presence of PTC (Fig 6D). The mRNA expression of nucleotide-binding oligomerization domain leucin rich repeat and pyrin domain containing protein 3 (NLRP3) increased in presence of the PTC (Fig 6E). The mRNA expression of the toll-like receptor 2 (TLR2) (Fig 6F), TLR4 (Fig 6G) and TLR9 (Fig 6H) decreased when exposed to PTC.

## Discussion

Since pigs showed early post-traumatic cardiac dysfunction and valvular insufficiency after multiple trauma [6], the aim of the present study was to investigate early structural and immunological local changes in cardiac tissue as well as the interplay with specific systemic alterations. Additionally, we were the first to study the impact of reamed vs. non-reamed femoral nailing with regard to the development of post-traumatic cardiac damage. In order to pinpoint the impact of systemic and local inflammatory conditions as present after trauma, additional *in vitro* experiments were conducted with murine cardiomyocytes. For this purpose, HL-1 cells were treated with a defined PTC, mimicking the inflammatory conditions after multiple trauma [23, 24].

Troponin is the most commonly used systemic marker for cardiac damage and was found to be an independent predictor for mortality after severe trauma [25, 26]. Troponin I and HFABP were previously shown to increase after experimental multiple trauma in pigs [6]. In the present study, the increased release of troponin I and HFABP from HL-1 cells into supernatant fluids after exposure to PTC indicates for myocardial damage in presence of inflammatory mediators and DAMPs *in vitro*.

We further observed cardiac structural changes, which might be an expression of the development of EMD after multiple trauma *in vivo*. Cardiac structural alterations (desmin and alpha-actinin) were previously demonstrated 72 h after multiple trauma and were linked to impaired cardiac function in pigs as well [6]. In the present report, we studied cardiac alterations after 6 h in separated left ventricle samples (luminal and superficial layers). The gap junction protein Cx43 was translocated from intercalated discs into the cytoplasm of the cardiomyocytes after multiple trauma in both parts of the left ventricle. This translocation of Cx43 was already demonstrated by our group in earlier studies after multiple trauma in pigs and mice [6, 27], after blunt chest trauma in rats [16], after new-born asphyxia in pigs [28] and during chronic psychosocial stress in mice [29]. The endocytosis of Cx43 has been linked to alterations in the electromechanical communication of CMs and is therefore associated with severe arrhythmia and cardiac dysfunction [30, 31]. Cell-cell communication through gap junctions such as Cx43 has been shown to partly prevent apoptosis in vitro [32]. Additionally, we were the first to analyse the expression of other relevant [33] cardiac gap junction proteins connexin 40 (Cx40) and connexin 45 (Cx45) in this study. Interestingly, a decrease of Cx40 mRNA expression occurred after multiple trauma with non-reamed medullary nailing in the superficial layers of left ventricles. Alterations in mRNA expression of Cx40, Cx43 and Cx45 were demonstrated previously after skeletal muscle crush injuries and were associated with regeneration and repair of damaged myofibers [34]. In presence of PTC, the mRNA expression of Cx43 increased in murine HL-1 cells, which might be mediated via toll-like receptor and NLRP3-inflammasome signalling as described previously [35, 36]. Therefore, the inflammatory response due fracture stabilization (reamed vs. non-reamed) might result in alterations of the cell-cell contact and therefore myocardial damage.

Moreover, we observed alterations in cardiac intermediate filament- and z-disc proteins 6 h after multiple trauma. While the protein expression of the z-disc protein alpha-actinin was not changed, the alpha-actinin mRNA expression significantly increased in pigs after reaming. The z-discs respond to strain and mechanical tension within the CMs and are therefore crucial for cardiac adaptions to haemodynamic demands [37]. Consequently, alterations in alpha-actinin expression might lead to the development of post-traumatic cardiac dysfunction.

Furthermore, the intermediate filament protein desmin was reduced in superficial layers of the left ventricles after multiple trauma with reamed medullary nailing. This is in contrast to findings of a previous study [6]. In our view this is most likely related to the shorter observation period in the current study compared to previous models.

In addition, a limitation of the study is the low animal number, which might bias the statistical analysis. The changes in desmin expression were described in the context of the so-called desminopathies, which impair the cardiac mechanical properties as well as the calcium homeostasis [38]. Mutations in the desmin gene have been linked to cardiomyopathy as well as to cardiac conduction diseases and to arrhythmia [39]. Desminopathies are associated with changes in the calcium amplitude of CMs and with alterations in the distribution and function of the ryanodine receptor [38]. Interestingly, increased levels of TNF have been shown to induce desmin cleavage via caspase 6, leading to a loss of desmin in intercalated discs, followed by aggregation in CMs [40]. Accordingly, in the present study presence of pro-inflammatory mediators induced an increased desmin mRNA expression in murine HL-1 cells *in vitro*. In a mouse model with desmin-related cardiomyopathy a remodelling of gap junction proteins such as Cx43 was observed, which is in accordance with the findings of the present study [41].

Summarized, cardiac structural alterations might contribute to EMD after multiple trauma in pigs. The alterations were intensified in pigs which were exposed to unreamed nailing. This makes it tempting to hypothesize an association between the development of cardiac dysfunction and the invasiveness of fracture fixation (reamed vs. unreamed). The structural cardiac alterations might be induced by inflammatory cardio-depressive cytokines such as TNF, IL-1β and IL-6 [15]. In the present study, we demonstrated a local increase of IL-1β and IL-6 in left ventricles after multiple trauma in animals which received reaming (reamed). The local cardiac increase of proinflammatory cytokines after trauma is in accordance with previous studies [6, 20]. The systemic increase of inflammatory mediators after reaming compared to non-reamed femoral nailing is discussed controversially in literature [12, 17, 42–46].

In the present report, IL-1β expression increased in left ventricles of pigs which received reaming. In rat CMs the presence of IL-1β induced a dysfunction of the cellular calcium balance by prolonging the calcium transient duration as well as the action potential, leading to an asynchronous calcium release during electrical stimulation, which was linked to arrhythmogenicity [47, 48]. In cultured cardiomyocytes, the presence of IL-6 led time-dependent to an increase of the iNOS protein amount and further presented a decrease of intracellular calcium [49].

Additionally, cytokines were described to activate the inducible nitric oxide synthase (iNOS) [50]. High iNOS activity results in enhanced nitrosative stress in the heart tissue, which is associated with impaired cardiac function after multiple trauma [6]. In the present study, nitrosative stress increased in left ventricles of pigs with reaming after multiple trauma, which is in accordance with our findings in previous studies [6].

Elevated local inflammation and nitrosative stress might be induced by systemic release of inflammatory mediators and DAMPs [51]. The current study demonstrates increased systemic HMGB-1 and extracellular histone levels 4 h and 6 h after trauma. Systemic release of HMGB-1 after trauma correlated with the injury severity as well as with the mortality of the patients [19, 52]. HMGB-1 was associated with the production of inflammatory cytokines like TNF, IL-1ß and IL-6 via Toll-like receptor-2,-4 and-9 [14, 53, 54]. Extracellular histones have been found to be cardio-depressive, leading to the development of posttraumatic as well as septic cardiomyopathy [55] Extracellular histones led dose- and time dependent to ROS production and increase of intracellular calcium, reduced dose-dependent the mitochondrial membrane potential and the ATP production, which resulted in reduced CM contraction, because of a lack of energy [56][57].

Finally, we investigated the expression of calcium handling proteins after multiple trauma *in vivo* as well as in presence of PTC *in vitro*. The RyR1 expression was downregulated 6 h after multiple trauma in pigs as well as in presence of PTC *in vitro*. Alterations in calcium handling proteins have earlier been linked to cardiac dysfunction after trauma [58]. Changes in these calcium handling proteins as well as a build-up of intracellular calcium might be induced by systemic inflammatory mediators and DAMPs [47, 55, 59]

### Summary

To summarize, local cardiac inflammation, enhanced nitrosative stress and impaired calcium handling, together with the systemic release of HMGB-1 and extracellular histones were associated with structural and functional alterations in cardiomyocytes and might therefore be linked to the development of EMD after multiple trauma *in vivo*. The detrimental effects were pronounced in pigs which received femoral reaming, underlining the interrelation of systemic inflammation and local cardiac damage. These findings are in accordance with earlier findings demonstrating enhanced local cardiac damage in pigs treated with more invasive early total care principles compared to less invasive damage control orthopaedics [6].

## Supporting information

S1 Fig Systemic amount of troponin after multiple trauma in pigs. Systemic levels of troponin I in serum presented as ng/ml at BL, 4 h and 6 h after trauma. BL presented as white bars, 4 h as medium grey bars and 6 h as dark grey bars. In each group n = 5. Results are significant (*) p<0.05. For statistical analysis one-way ANOVA was used. Graphical presentation as mean ± SEM.
(PDF)

S2 Fig Summarizing result table. (PDF)

S1 Raw images (PDF)

S1 Table RT-qPCR primer. (DOCX)

## List of abbreviations

ARDS: Acute respiratory distress syndrome
BL: Baseline
C3a/ 5a: Complement component 3a/ 5a
CM: Cardiomyocyte
Cx 40/ 43/45: Connexin 40/ 43/ 45
DAMPs: Damage associate, d molecular patterns
EMD: Early myocardial damage
FiO_2_: Fraction of inspired oxygen
GJA1: Gap junction alpha-1 protein (synonym for Cx43)
HFABP: Heart fatty acid binding protein
HMGB-1: High Mobility Group Box Protein-1
HRP: Horseradish peroxidase
IF: Immunofluorescence
IgG: Immunoglobulin G
IHC: Immunohistochemistry
IL-1ß: -6, -8, Interleukin 1ß, 6, 8
LV: Left ventricle
iNOS: inducible nitric oxide synthase
ISS: Injury severity score
MAP: Mean arterial pressure
mRNA: messenger RNA
NCX: Sodium-calcium exchanger
NLRP3: Nucleotide-binding oligomerization domain leucin rich repeat and pyrin domain containing protein 3
PTC: Polytrauma-cocktail
RT-qPCR: Real-time quantitative PCR
RyR1: Ryanodine receptor 1
SEM: Standard error of the mean
SERCA: sarcoplasmic/endoplasmic reticulum ATPase
s-ICAM-1: Soluble intercellular adhesion molecule 1
TLR2: -4, -9, Toll-like receptor 2, 4, 9
TNF alpha: Tumor necrosis factor alpha
