# Supplementary figures and images for: Structural alterations and inflammation in the heart after multiple trauma followed by reamed versus non-reamed femoral nailing
Source: PLoS One. 2020 Jun 25;15(6):e0235220. doi: 10.1371/journal.pone.0235220 (PMC7316303; doi:10.1371/journal.pone.0235220)

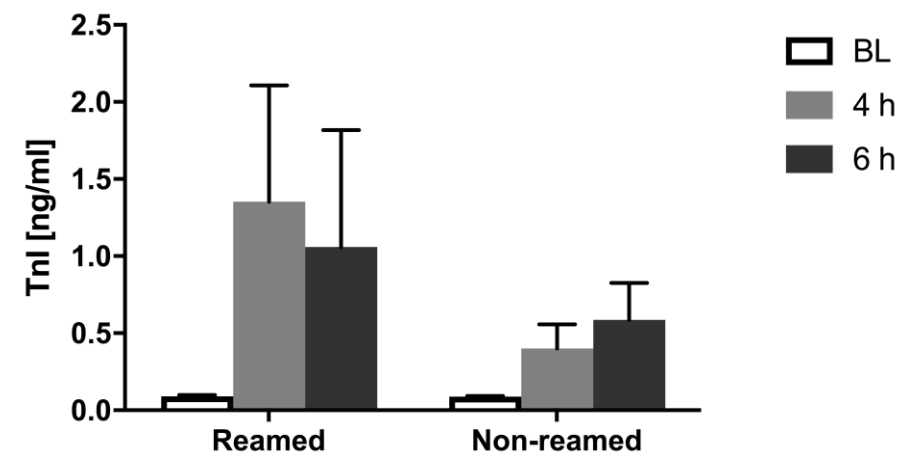

Supplement: S1 Fig — Systemic levels of troponin I in serum presented as ng/ml at BL, 4 h and 6 h after trauma. BL presented as white bars, 4 h as medium grey bars and 6 h as dark grey bars. In each group n = 5. Results are significant (*) p<0.05. For statistical analysis one-way ANOVA was used. Graphical presentation as mean ± SEM. (PDF) [file pone.0235220.s001.pdf]

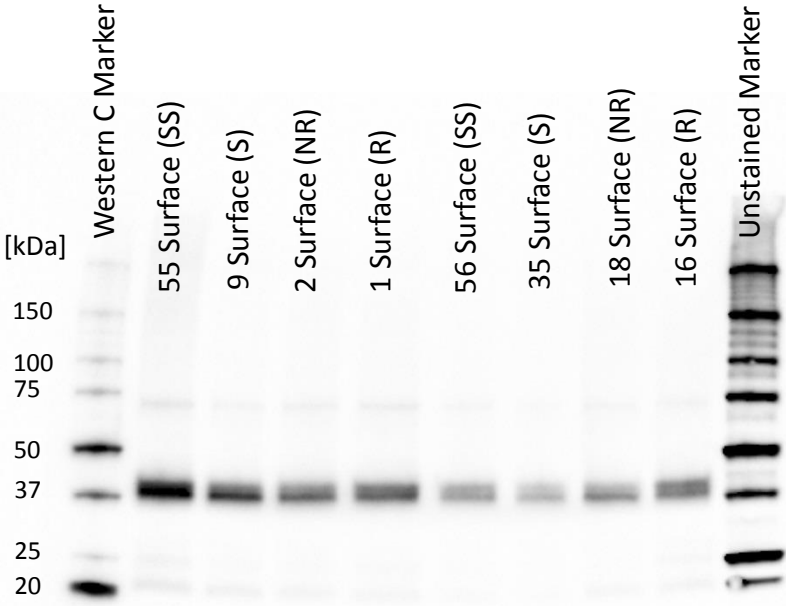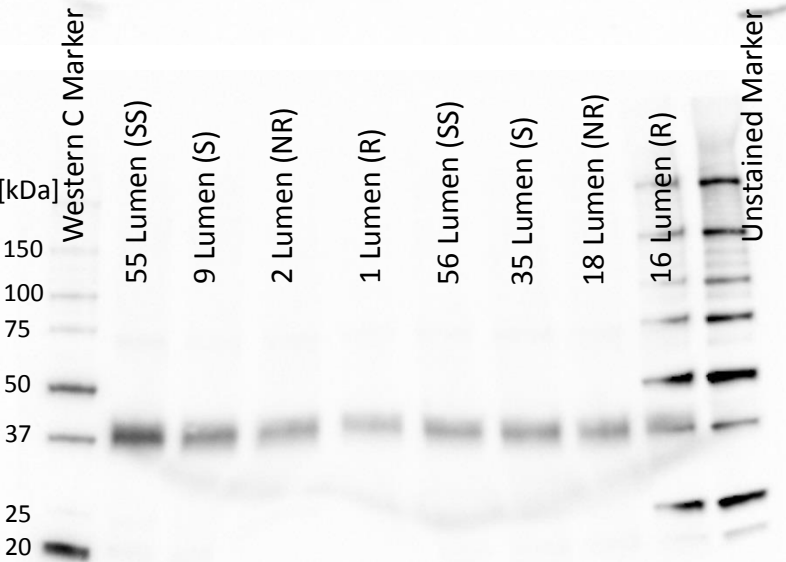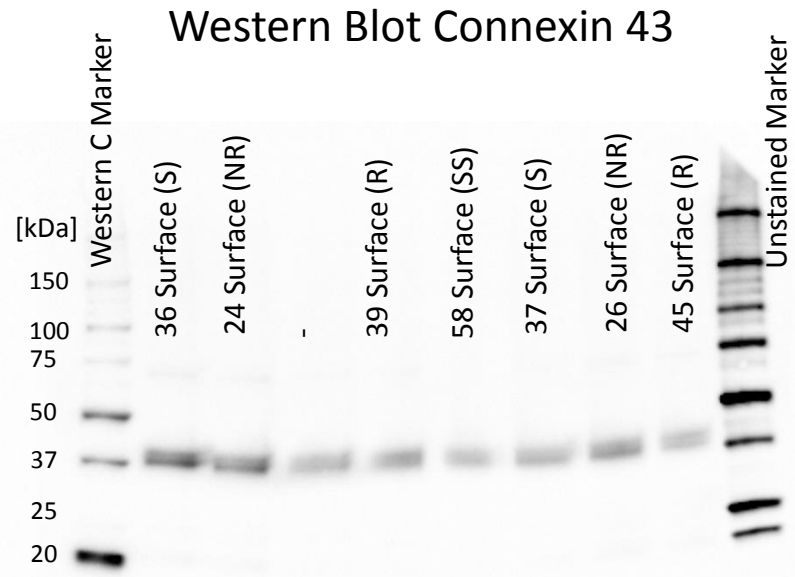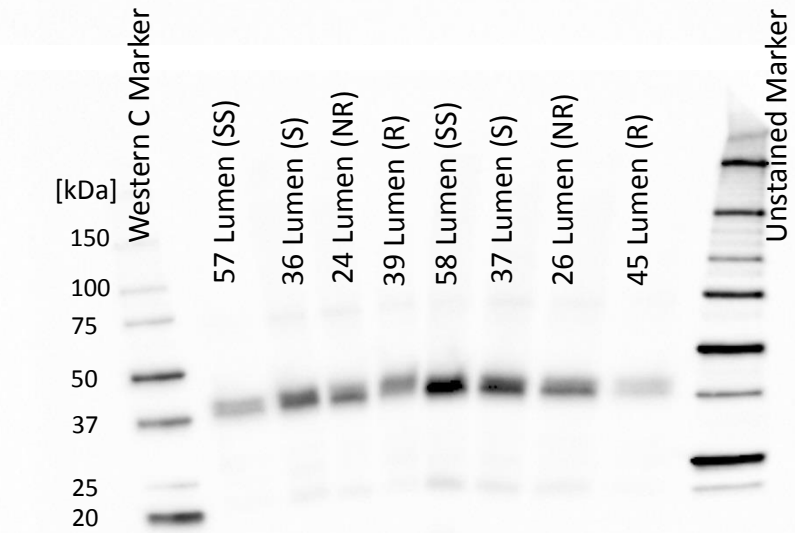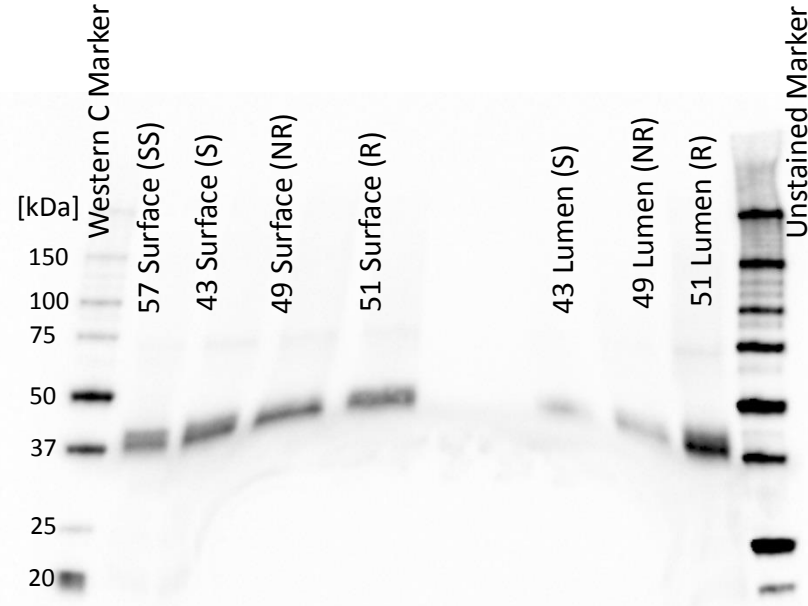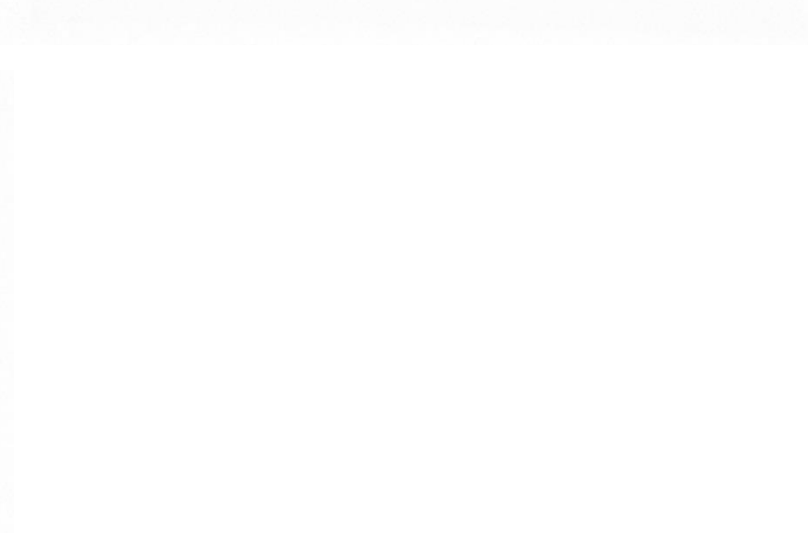

Western Blot Interleukin-6

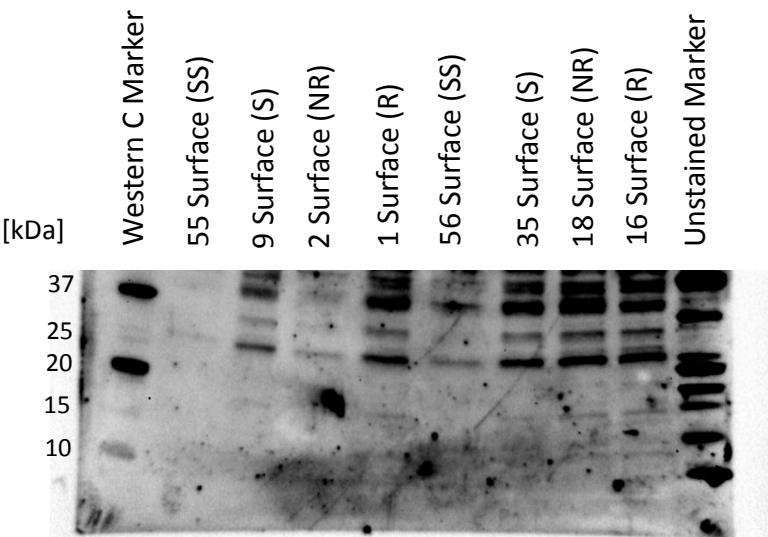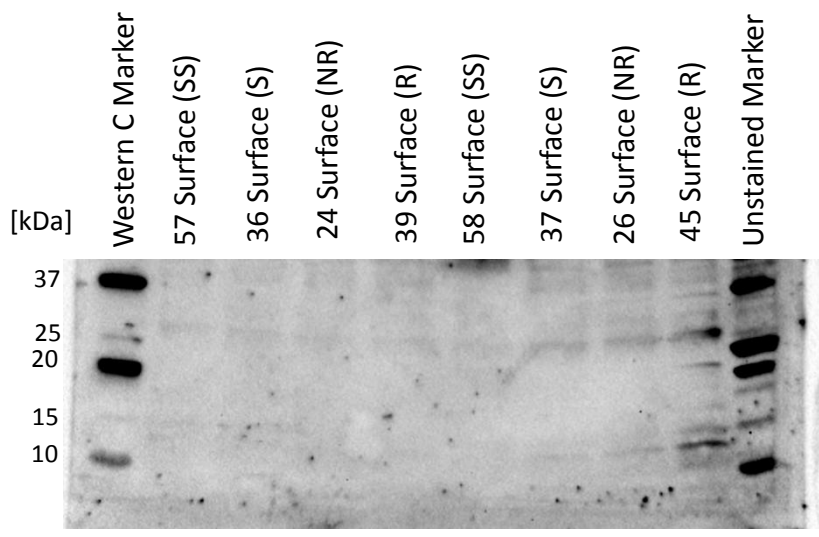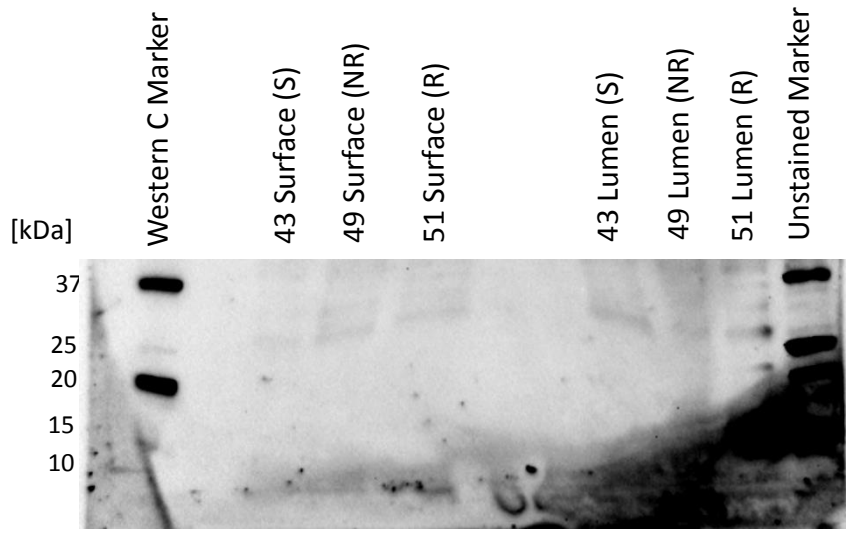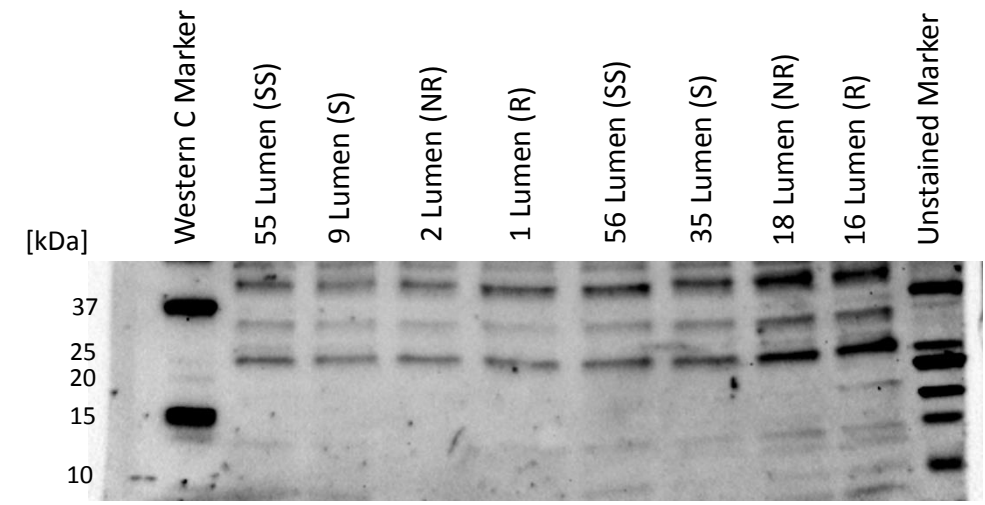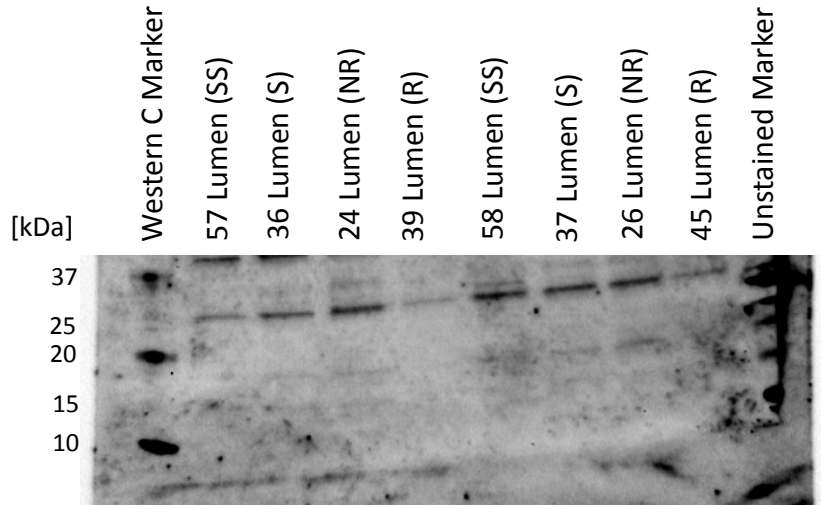

Supplement: S1 Raw images — (PDF) [file pone.0235220.s003.pdf]
