# Supplementary material for: Structural alterations and inflammation in the heart after multiple trauma followed by reamed versus non-reamed femoral nailing
Source: PLoS One. 2020 Jun 25;15(6):e0235220. doi: 10.1371/journal.pone.0235220 (PMC7316303; doi:10.1371/journal.pone.0235220)
Supplement: S2 Fig — (PDF) [file pone.0235220.s002.pdf]

	sham	reamed	non-reamed
Cx43 (mRNA)	-	-	reduced at the surface layers of the left ventricle compared to sham
Cx43 (protein)	no difference	no difference	no difference
Cx40 (mRNA)	-	-	reduced at the surface layers of the left ventricle compared to sham
Cx45 (mRNA)	no difference	no difference	no difference
Alpha-actinin (protein)	no difference	no difference	no difference
Alpha-actinin (mRNA)	-	reduced at the luminal layer of the left ventricle compared to sham and non-reamed	-
Desmin (protein)	no difference	no difference	no difference
Desmin (mRNA)	-	reduced at the superficial layers of the left ventricle compared to sham	-
IL-1ß (mRNA)	no difference	no difference	no difference
IL-6 (protein)	no difference	no difference	no difference
IL-6 (mRNA)	-	increased at the superficial layer of the left ventricle compared to non-reamed	-
Nitrotyrosine (protein)	-	increased at the superficial layer of the left ventricle compared to sham	-

HMGB-1 (systemically)	-	increased 6h after trauma	increased 4 and 6h after trauma
Histones (systemically)	-	increased 6h after trauma	increased 4h after trauma
RyR1 (mRNA)	-	-	reduced at luminal layer of the left ventricle compared to sham
SERCA (mRNA)	-	increased at luminal layer of the left ventricle compared to non-reamed	-
NCX (mRNA)	no difference	no difference	no difference
