# Supplementary material for: Structural alterations and inflammation in the heart after multiple trauma followed by reamed versus non-reamed femoral nailing
Source: PLoS One. 2020 Jun 25;15(6):e0235220. doi: 10.1371/journal.pone.0235220 (PMC7316303; doi:10.1371/journal.pone.0235220)
Supplement: S1 Table — (DOCX) [file pone.0235220.s004.docx]

**RT-qPCR primer**

| mouse aActinin (for)  mouse aActinin (rev) | 5’-AACCTGGCCATGGAAATAGCA-3’  5’-ATCGGGTTTGGGAGTGTTGA-3’ |
| --- | --- |
| mouse Cx43 (for)  mouse Cx43 (rev) | 5‘-GGCCACAGGTGAGACCATTA-3‘  5‘-CGGCCATCGTTGTTCTTGTC-3‘ |
| mouse Desmin (for)  mouse Desmin (rev) | 5‘-CTCGGATATCACACCCAGCC-3‘  5‘-CACAAAGGGGTGATCGGTGA-3‘ |
| mouse GAPDH (for)  mouse GAPDH (rev) | 5‘CTTCAACAGCAACTCCCACTCTTCC3‘  5‘-GGTGGTCCAGGGTTTCTTACTCC-3‘ |
| mouse HFABP (for)  mouse HFABP (rev) | 5’-TGACCGGAAGGTCAAGTCAC-3’  5’-TTAGTGTTGTCTCCTGCCCG-3’ |
| mouse NCX (for)  mouse NCX (rev) | 5‘-CCTTGTGCATCTTAGCAATG-3‘  5‘-TCTCACTCATCTCCACCAFA-3‘ |
| mouse NLRP3 (for)  mouse NLRP3 (rev) | 5‘-GCTGCTCAGCTCTGACCTCT-3‘  5‘-AGGTGAGGCTGCAGTTGTCT-3‘ |
| mouse RyR1 (for)  mouse RyR1 (rev) | 5‘-AATGGCCAAGGCAGGAGTGG-3‘  5‘-TCAAGGATGTCTGCACGGAGT-3‘ |
| mouse SERCA (for)  mouse SERCA (rev) | 5‘-TACCTGGAACAACCCGCAAT-3‘  5‘-CAGAGCACAGATGGTGGCTAAC-3‘ |
| mouse TLR2 (for)  mouse TLR2 (rev) | 5‘-GAAACCTCAGACAAAGCGTCA-3‘  5‘-ACAGCGTTTGCTGAAGAGGA-3‘ |
| mouse TLR4 (for)  mouse TLR4 (rev) | 5‘-GGACTCTGATCATGGCACTGT-3‘  5‘-GGAACTACCTCTATGCAGGGAT-3‘ |
| mouse TLR9 (for)  mouse TLR9 (rev) | 5‘-GAGAGACCCTGGTGTGGAAC-3‘  5‘-CCTTCGACGGAGAACCATGT-3‘ |
| mouse Troponin I (for)  mouse Troponin I (rev) | 5‘-GATGCGGCTGGGGAACC-3‘  5‘-ACTTTTTCTTGGCGTGTGGC-3‘ |

| pig aActinin (for)  pig aActinin (rev) | 5’-CCGCATCATGAGCATTGTGG-3’  5’-CCGTCTCACGGGACATGAAA-3’ |
| --- | --- |
| pig Connexin40 (for)  pig Connexin40 (rev) | 5’-CTGCCACCACATGTCATCCT-3’  5’-GCTGGACAAACAGCCAAAGG-3’ |
| pig Connexin43 (for)  pig Connexin43 (rev) | 5’-GTTTCCTCTCTCGTCCCACG-3’  5’-GTTCAAGGCGAGAGACACCA-3’ |
| pig Connexin45 (for)  pig Connexin45 (rev) | 5’-CCAGATCATCCTGGTCGCAA-3’  5’-AGCTGCCTTCTTGTCTGCTT-3’ |
| pig Desmin (for)  pig Desmin (rev) | 5’-GCCGGATCAACCTCCCTATC-3’  5’-ACCTCAGAACCCCTTTGCTC-3’ |
| pig GAPDH (for)  pig GAPDH (rev) | 5‘-GAGTGAACGGATTTGGCC-3‘  5‘-AAGGGGTCATTGATGGCGAC-3‘ |
| pig IL-1β (for)  pig IL-1β (rev) | 5‘-CAGCCAGTCTTCATTGTTCAGG-3‘  5‘GGTCATTATTGTTGTCACCGTAGT3‘ |
| pig IL-6 (for)  pig IL-6 (rev) | 5‘-CCCACCAGGAACGAAAGAGA-3‘  5‘-TGAAGGCGCTTGTGGAGAG-3‘ |
| pig NCX (for)  pig NCX (rev) | 5’GATGGAACGTGGAATATCAGTGC3’  5’-TTCCTCCACAGTCAGCTTCC-3’ |
| pig SERCA (for)  pig SERCA (rev) | 5’-CTGCTCGTTCTCGGCATGTA-3’  5’-CCAGATCACCAGCGGCATTA-3‘ |
| pig RyR1 (for)  pig RyR1 (rev) | 5’-AACTGCTACATGGTGTGGGG-3’  5’-AGCCGATGACAAGGTCTGTG-3‘ |
